# *ZmCCT* regulates photoperiod-dependent flowering and response to stresses in maize

**DOI:** 10.1186/s12870-021-03231-y

**Published:** 2021-10-06

**Authors:** Huihui Su, Jiachen Liang, Salah Fatouh Abou-Elwafa, Haiyang Cheng, Dandan Dou, Zhenzhen Ren, Jiarong Xie, Zhihui Chen, Fengran Gao, Lixia Ku, Yanhui Chen

**Affiliations:** 1 Synergetic Innovation Center of Henan Grain Crops and National Key Laboratory of Wheat and Maize Crop Science, Henan Agricultural University, Zhengzhou, 450046 Henan China; 2 Agronomy Department, Faculty of Agriculture, Assiut University, Assiut, Egypt

**Keywords:** DAP-Seq, Maize, Flowering time, Circadian period, Stress response, *ZmCCT*

## Abstract

**Background:**

Appropriate flowering time is very important to the success of modern agriculture. Maize (*Zea mays* L.) is a major cereal crop, originated in tropical areas, with photoperiod sensitivity. Which is an important obstacle to the utilization of tropical/subtropical germplasm resources in temperate regions. However, the study on the regulation mechanism of photoperiod sensitivity of maize is still in the early stage. Although it has been previously reported that *ZmCCT* is involved in the photoperiod response and delays maize flowering time under long-day conditions, the underlying mechanism remains unclear.

**Results:**

Here, we showed that *ZmCCT* overexpression delays flowering time and confers maize drought tolerance under LD conditions. Implementing the Gal4-LexA/UAS system identified that *ZmCCT* has a transcriptional inhibitory activity, while the yeast system showed that *ZmCCT* has a transcriptional activation activity. DAP-Seq analysis and EMSA indicated that *ZmCCT* mainly binds to promoters containing the novel motifs CAAAAATC and AAATGGTC. DAP-Seq and RNA-Seq analysis showed that *ZmCCT* could directly repress the expression of *ZmPRR5* and *ZmCOL9*, and promote the expression of *ZmRVE6* to delay flowering under long-day conditions. Moreover, we also demonstrated that *ZmCCT* directly binds to the promoters of *ZmHY5*, *ZmMPK3*, *ZmVOZ1* and *ZmARR16* and promotes the expression of *ZmHY5* and *ZmMPK3*, but represses *ZmVOZ1* and *ZmARR16* to enhance stress resistance. Additionally, *ZmCCT* regulates a set of genes associated with plant development.

**Conclusions:**

*ZmCCT* has dual functions in regulating maize flowering time and stress response under LD conditions. *ZmCCT* negatively regulates flowering time and enhances maize drought tolerance under LD conditions. *ZmCCT* represses most flowering time genes to delay flowering while promotes most stress response genes to enhance stress tolerance. Our data contribute to a comprehensive understanding of the regulatory mechanism of *ZmCCT* in controlling maize flowering time and stress response.

**Supplementary Information:**

The online version contains supplementary material available at 10.1186/s12870-021-03231-y.

## Background

Maize (*Zea mays* L.), which was domesticated in Southern Mexico roughly 9000 years ago from Balsas teosinte and spread through North and South America before the arrival of Europeans, requires short-day conditions to flower [1]. Tropical maize genotypes are generally sensitive to long-days condition. Flowering time is one of the most important traits that determines plant adaptability to environmental cues [2, 3]. Several genes implicated in maize floral transition have been identified and functionally characterized including the CCT domain-containing genes *ZmCCT*, *ZmCCT9*, and the maize *CONSTANS LIKE 3* (*ZmCOL3*), *Zea mays* MADS-box genes *ZMM4* and *ZmMADS69*, the circadian clock component genes *ZmCCA1* and *ZmCCA1a*, *Zea mays CENTRORADIALIS 8* (*ZCN8*), and *Zea mays* NF-Y transcriptional factor gene *ZmNF-YA3*. CCT domain genes play an important role in flowering time regulation in maize [4]. *ZmCOL3* appears to affect flowering time primarily under long days (LD) conditions. *ZmCOL3* represses flowering time by activating the expression of *ZmCCT* [4], which functions as a flowering repressor in maize under LD conditions [5, 6]. A Harbinger-like transposable element that tropical SD maize germplasms do not have, acts in *cis* to promote flowering under LD conditions by repressing the expression of *ZmCCT9* [7]. Under LD conditions, *ZmCCT9* delays flowering time by negative regulation of the florigen gene *ZCN8* [7]. *ZCN8* underlies the major flowering time quantitative trait locus *qDTA8* that is involved in photoperiod sensitivity [8, 9]. The MADS-box transcription factors play important roles in flower development and plant inflorescence [10]. *ZmMADS69* suppresses the expression of the flowering repressor *ZmRap2.7*, thereby promotes the expression of *ZCN8* and causing early flowering [10]. *ZMM4* promotes floral transition, and the maize transgenic plants overexpress *ZMM4* flowered earlier than the wild type [11]. Plants adjust daily changes and seasonal changes by the circadian clock, an endogenous mechanism that controls a wide range of biological processes [12, 13]. *ZmCCA1*, an ortholog of *AtCCA1*, is expressed in a rhythmic pattern, and the overexpression of *ZmCCA1* in *A. thaliana* delayed flowering time [14]. Shi et al. [15] showed that *ZmCCA1a* is likely to be an important component of the circadian clock in maize, and flowering time was delayed in the *ZmCCA1a*-overexpressing *A. thaliana* lines under LD conditions. NF-Ys are widespread in eukaryotes. Several studies showed that NF-Ys play an important role in flowering time regulation [16–19]. In *Arabidopsis*, the *nf-yc3/yc4/yc9* triple mutant produced almost twice as many total leaves before flowering compared to the wild type under LD but not SD conditions, indicating that *NF-YC3*, *NF-YC4* and *NF-YC9* genes are involved in photoperiod-dependent flowering regulation [16]. The overexpression of the *OsNF-YC2* could rescue the late-flowering phenotype of the Arabidopsis *nf-yc3/yc4/yc9* triple mutant [18]. *ZmNF-YA3* encodes an NF-YA subunit in maize, and the *zmnf-ya3* mutant showed delayed flowering under LD conditions, whereas there was no significant difference in flowering time compared to the WT under SD conditions [19].

A growing number of evidence showed that photoperiod responsive genes play important roles in plant response to biotic and abiotic stresses [20–23]. Tian et al. [20] showed that transgenic *Arabidopsis* plants overexpressing *ZmCCA1.1* exhibited higher tolerance to drought stress. *AtPRR7*, a central component of the *Arabidopsis* clock, negatively regulates stress responses via direct regulation of drought- and abscisic acid-responsive genes [21]. Wang et al. [22] identified new genes responsible for *R*-gene-mediated resistance to downy mildew in *Arabidopsis*, and they are controlled by the circadian rhythm regulator *CCA1*. Abscisic acid, osmotic and salt stresses could induce the expression of *AtCOL4* [23], which is an essential regulator of abiotic stress tolerance in *Arabidopsis*. Su et al. [19] showed that *ZmNF-YA3* enhances stress resistance in maize under LD conditions rather than positive regulation of flowering time. The relative water contents (RWC) of *zmnf-ya3* mutant was significantly lower than WT plants after 1 and 4 days of drought and heat stress induction under LD conditions, respectively. *ZmNF-YA3* improves plant tolerance to drought and heat stresses via direct regulation of the expression of the *bHLH92*, *FAM* and JA activator gene *MYC4* [19].

A previous study revealed that *ZmCCT* plays a negative role in regulating flowering time in maize [5, 6]. Hung et al. [5] showed that the expression of *ZmCCT* alleles from diverse teosintes is higher than that from temperate maize and confer delayed flowering phenotype under LD conditions. In early-flowering maize, Yang et al. [6] detected a CACTA-like transposable element within the *ZmCCT* promoter that markedly reduces flowering time. The CACTA-like transposable element represses the expression of *ZmCCT* to reduce photoperiod sensitivity. Consequently, maize could flower early under LD conditions. *ZmCCT* is a homolog of the rice photoperiod response regulator gene *Ghd7*. *Ghd7* is diurnally expressed, and the expression of *Ghd7* was much higher under LD compared to SD conditions. *Ghd7* is expressed in young tissues, such as developing leaves, apical meristem and leaf sheaths [24]. The teosinte *ZmCCT* allele showed a diurnal expression pattern under LD conditions, with higher transcription levels observed in the light [6]. *ZmCCT* is expressed in the leaves and shoot apical meristem at 3–7 leaf stages, plant ovule and pollen [25–27]. Enhanced expression of *Ghd7* under LD conditions delays heading date in rice [24]. The expressions of *Hd3a* and *Ehd1* were suppressed by *Ghd7* under LD conditions [24]. Ku et al. [25] showed that the RWC of the HZ4-NIL containing *ZmCCT*-associated QTL was remarkably higher than that of HZ4 after drought and heat stress induction. The co-expression analysis and the diurnal rhythms of stress response-related genes suggest *ZmCCT* as a crucial functional crosslink linking photoperiod with stress tolerance responses under LD conditions [25]. However, the molecular mechanisms of ZmCCT in photoperiod-dependent flowering time regulation and response to biotic/abiotic stresses in maize are still ambiguous. Here we showed that overexpression of *ZmCCT* delays flowering time and confers drought tolerance in maize under LD conditions. Implementing the Gal4-LexA/UAS system revealed that *ZmCCT* has a transcriptional inhibitory activity, while the yeast system showed that *ZmCCT* has a transcriptional activation activity. The DAP-Seq assay and EMSA showed that *ZmCCT* binds to the promoters containing the motifs CAAAAATC and AAATGGTC. Moreover, DAP-Seq and RNA-Seq analyses showed that *ZmCCT* regulates genes implicated in photoperiod-dependent flowering time regulation, stress response and plant development. This work contributes to a comprehensive understanding of the molecular mechanism of photoperiod affecting maize flowering time and stress response.

## Results

### Phenotypic variations in flowering time and drought stress tolerance under LD conditions

**Fig. 1:**
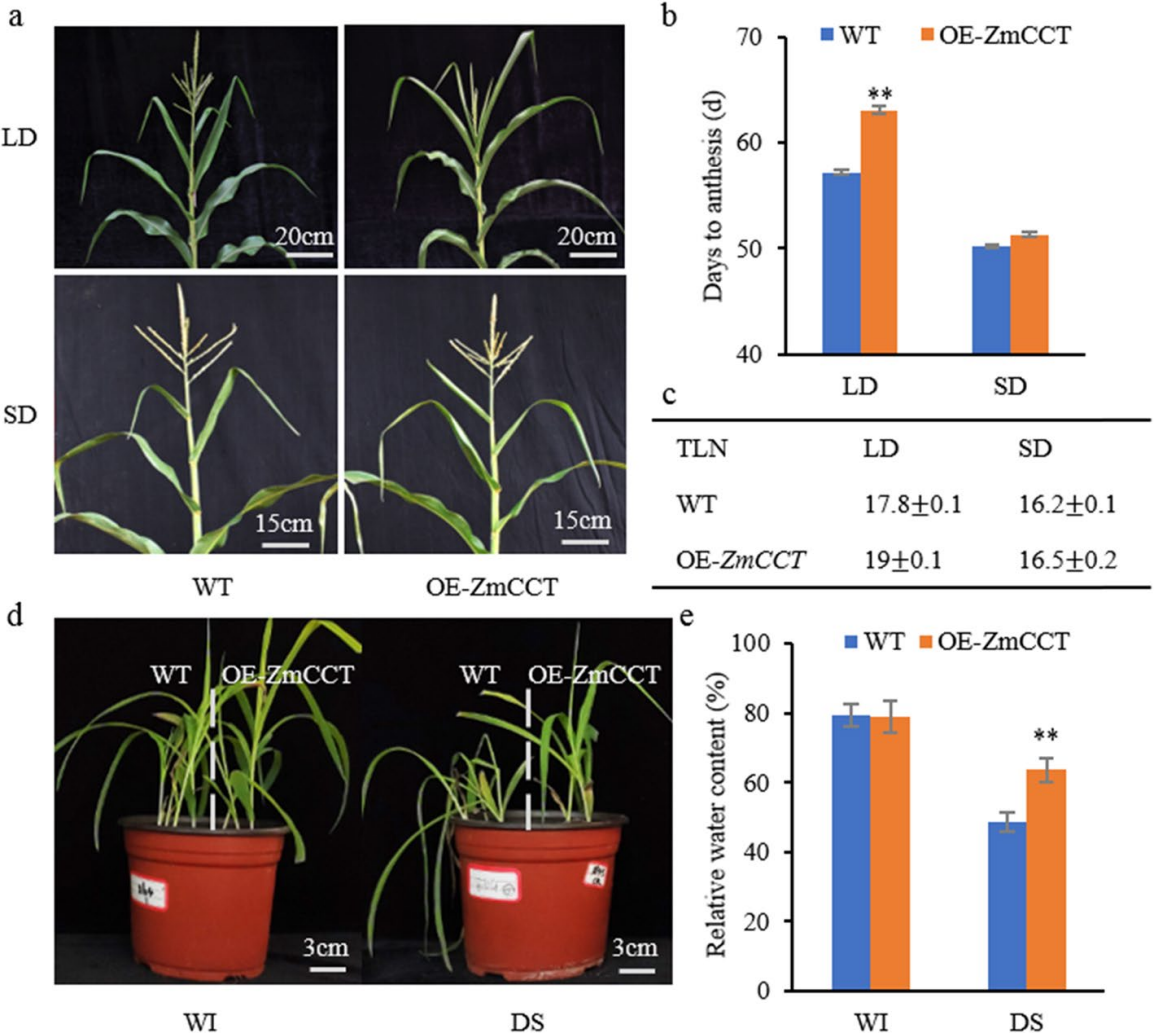
Phenotypic evaluation of flowering time and stress response of *ZmCCT.* **a**) Phenotypes of the WT and OE-*ZmCCT* lines under LD and SD conditions at the V6 growth stage. Bar = 15 or 20 cm. **b**) Flowering time of the WT and OE-*ZmCCT* lines under SD and LD conditions. Seven T_2_ transgenic lines were evaluated. Data are shown as an average (*n* = 3, ±SD, ** *P* < 0.01). **c**) The total leaf number (TLN) of the WT and OE-*ZmCCT* lines under LD and SD conditions. Sixteen plants were used from each line. Data are shown as an average ± SD. **d**) Phenotypes of the WT and OE-*ZmCCT* lines under well-irrigated (WI) and drought-stressed (DS) treatments under LD conditions. Bar = 3 cm. **e**) Relative water content (RWC) of the WT and OE-*ZmCCT* lines under well-irrigated (WI) and drought-stressed (DS) treatments. Two T_2_ transgenic lines were used. (Data are shown as an average (*n* = 3) ± SD, ** *P* < 0.01)

Seven T_0_ transgenic plants were obtained from three independent transgenic events. Compared to the WT plants, T_2_ families of transgenic plants exhibited 5.9 days delayed anthesis under LD conditions (Fig. 1a, b). Under SD conditions, no significant differences were observed between OE-*ZmCCT* and the WT plants in the number of days to anthesis (Fig. 1a, b). The total leaf number (TLN) of the OE-*ZmCCT* was higher than WT plants under LD conditions (Fig. 1c). These results suggest that overexpression of *ZmCCT* delays maize flowering under LD conditions. Moreover, the OE-*ZmCCT* and WT plants exhibited significant differences in response to drought stress under LD conditions (Fig. 1d). The relative water content (RWC) was estimated to investigate the response of the OE-*ZmCCT* and WT plants to drought stress at the physiological level. The RWC of OE-*ZmCCT* plants was significantly higher than that of the WT plants after drought stress induction (Fig. 1e). These results indicate that *ZmCCT* has dual functions in regulating maize flowering time and stress response under LD conditions.

### RNA-Seq identification of genes affected by *ZmCCT*

**Fig. 2:**
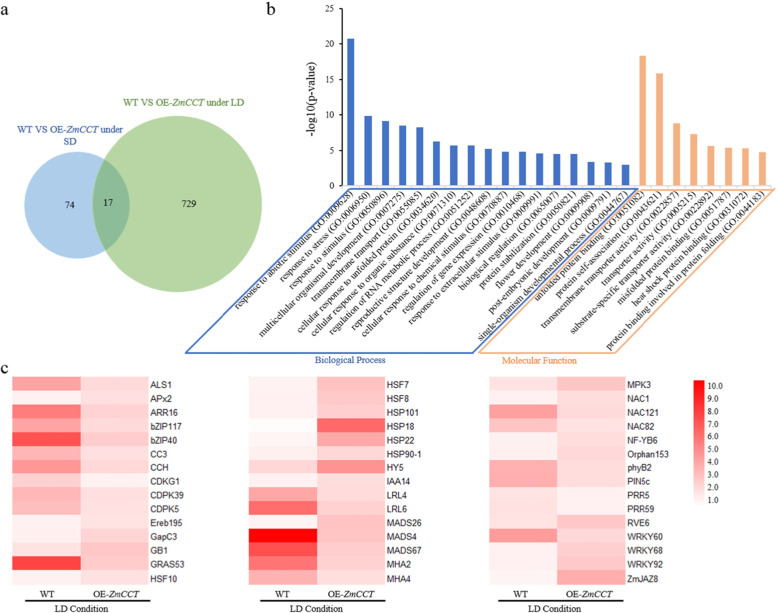
Transcriptomic analysis of *ZmCCT*. **a)** Venn diagram shows DEGs identified between the WT and OE-*ZmCCT* plant under SD and LD conditions. **b)** GO annotation of DEGs between the WT and OE*-ZmCCT* plant under LD conditions*.* **c)** Heat map shows differential expression of genes related to flowering time and stress response. The FPKM was employed used to make heat map

To understand the regulatory network of *ZmCCT* in response to photoperiod-mediated flowering, RNA-Seq analysis was conducted using total RNA extracted from leaves of the wild type (WT) and *ZmCCT* over-expressing plants (OE-*ZmCCT*) at V6 growth stage under both SD and LD conditions. On average, 21.4–27.3 million 150-nt clean reads were generated for each cDNA library (12 cDNA libraries in total), and ~ 83% of the reads were uniquely mapped to the maize reference genome V4 (Additional file 1). To dissect the changes in the gene expression in response to LD conditions, differentially expressed genes (DEGs) between the WT and OE*-ZmCCT* plants under SD and LD conditions were investigated. Genes showed significant changes in expression (> two-fold, i.e., log2 foldchange > 1 or log2 foldchange <− 1, padj < 0.05) were selected for further analysis. Accordingly, 91 and 746 genes DEGs were identified under SD and LD conditions, respectively (Fig. 2a). To validate the differential expression observed from RNA-Seq analysis between the WT and OE*-ZmCCT* under SD and LD conditions, RT-qPCR on 20 DEGs under LD conditions was performed (Additional file 2). RT-qPCR of those 20 DEGs showed similar levels of differential expression patterns observed from the RNA-Seq analysis, indicating the reliability of RNA-Seq analysis in the identification of DEGs (Additional file 3, Fig. S1). The agriGO v2.0 analysis toolkit was employed to perform Gene Ontology (GO) enrichment analysis [28]. Out of the 746 DEGs identified between the WT and OE*-ZmCCT* under LD conditions, 599 DEGs were functionally annotated (Fig. 2a, b). The GO term enrichment analysis of the 599 DEGs revealed that these genes are mainly involved in cellular process (GO:0050794, *p* value = 5.2 × 10^− 4^), response to stimulus (GO:0050896, *p* value = 8.00 × 10^− 10^), metabolic process (GO:0019219, *p* value = 1.2 × 10^− 6^), biological regulation (GO:0065007, *p* value = 3.4 × 10^− 5^), developmental process (GO:0044767, *p* value = 0.001), flower development (GO:0009908, *p* value = 4.1 × 10^− 4^). The most significant subcategory was “response to abiotic stimulus” (GO:0009628, *p* value = 2.00 × 10^− 21^). The other interesting significant subcategories are “response to stimulus” and “flower development”. Heatmap showed some differentially expressed genes between the WT and OE-*ZmCCT* under LD conditions (Fig. 2c, Additional file 4).

### *ZmCCT* has both transcriptional activation and inhibitory activities

**Fig. 3:**
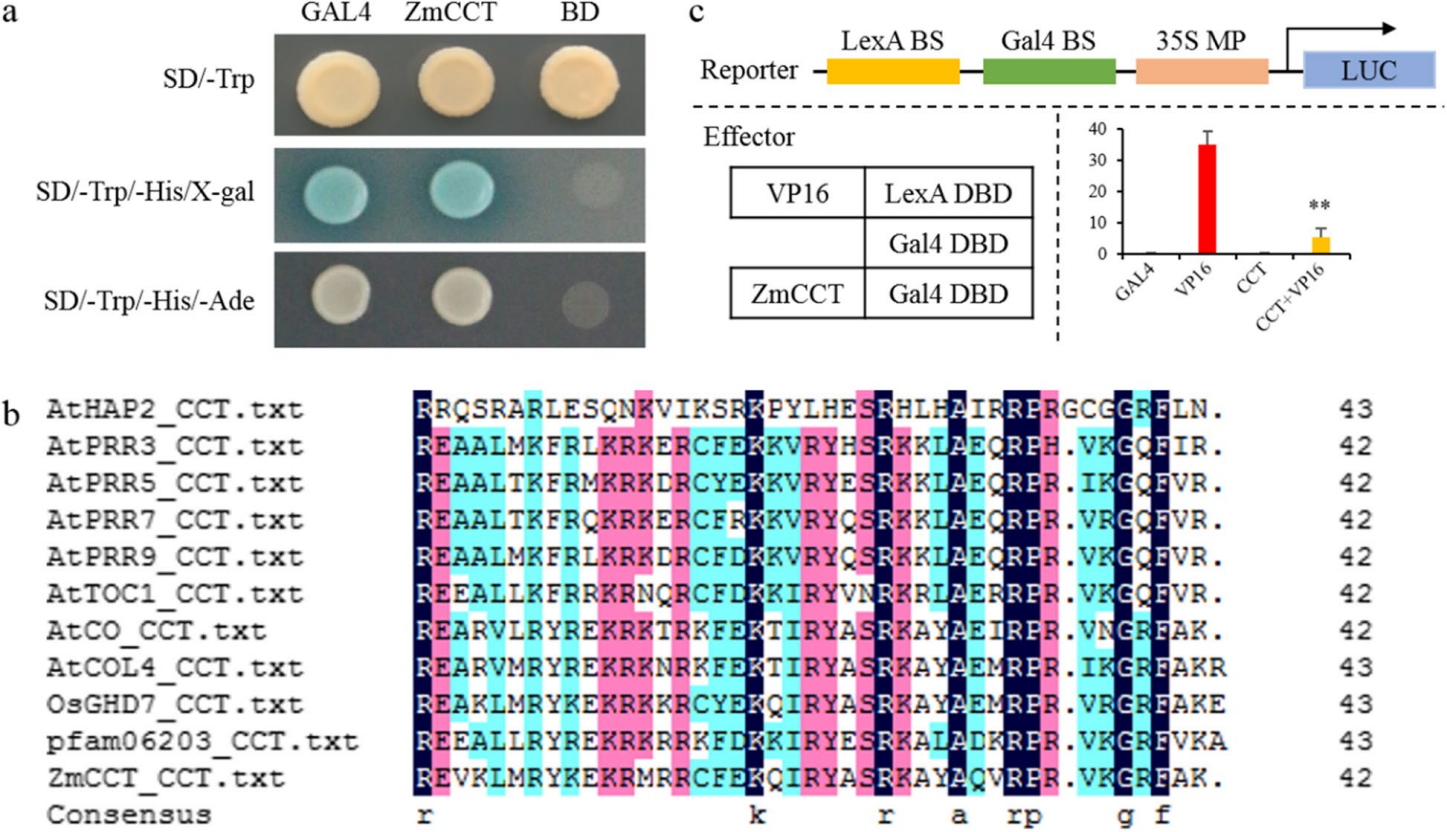
Analysis of transcriptional activity of ZmCCT protein. **a)** Transactivation analysis of *ZmCCT* fused to the GAL4 DNA-binding domain in yeast. The fusion vectors pGBKT7-ZmCCT, pGBKT7-Gal4 (GAL4) and pGBKT7 (BD) were respectively transformed into the yeast competent cells. Firstly, the deficient medium SD/−Trp was screened, and then the positive monoclones were selected for verification in the deficient medium SD/−Trp/−His/X-gal and SD/−Trp/−His/−Ade. GAL4 was the positive control and BD was the negative control. **b)** Sequence alignment of CCT-domain from *ZmCCT*, *OsGHD7* and eight Arabidopsis CCT-domain containing gene. The alignment was performed using DNAMAN. **c)** Determination of *ZmCCT* transcriptional activity. The reporter (upper) and effector constructs (left) were co-transformed into *N. benthamiana* leaves, and the expression level of the reporter gene was determined (right) (*n* = 5, ±SD, ** P < 0.01). VP16: VP16-LexA fusion protein; Gal4: Gal4 DNA binding domain (DBD); CCT: full-length *ZmCCT* fused to Gal4 DBD

To investigate whether *ZmCCT* can act as a gene expression promotor or repressor, transactivation analysis of *ZmCCT* in yeast was performed. *ZmCCT* exhibits obvious transcriptional activation activity (Fig. 3a). However, *ZmCCT* encodes a CCT-domain protein (Fig. 3b) that has been reported to have a transcriptional inhibitory activity [21, 29]. To confirm that, the Gal4-LexA/UAS system that analyzes the proteins for positive or negative transcriptional potential was implemented. *ZmCCT* significantly decreased the expression of the reporter gene (Fig. 3c), indicating that *ZmCCT* has a transcriptional inhibitory activity. Altogether, *ZmCCT* is likely to have both transcriptional activation and inhibitory activities.

### DAP-Seq identifies *ZmCCT* target genes

**Fig. 4:**
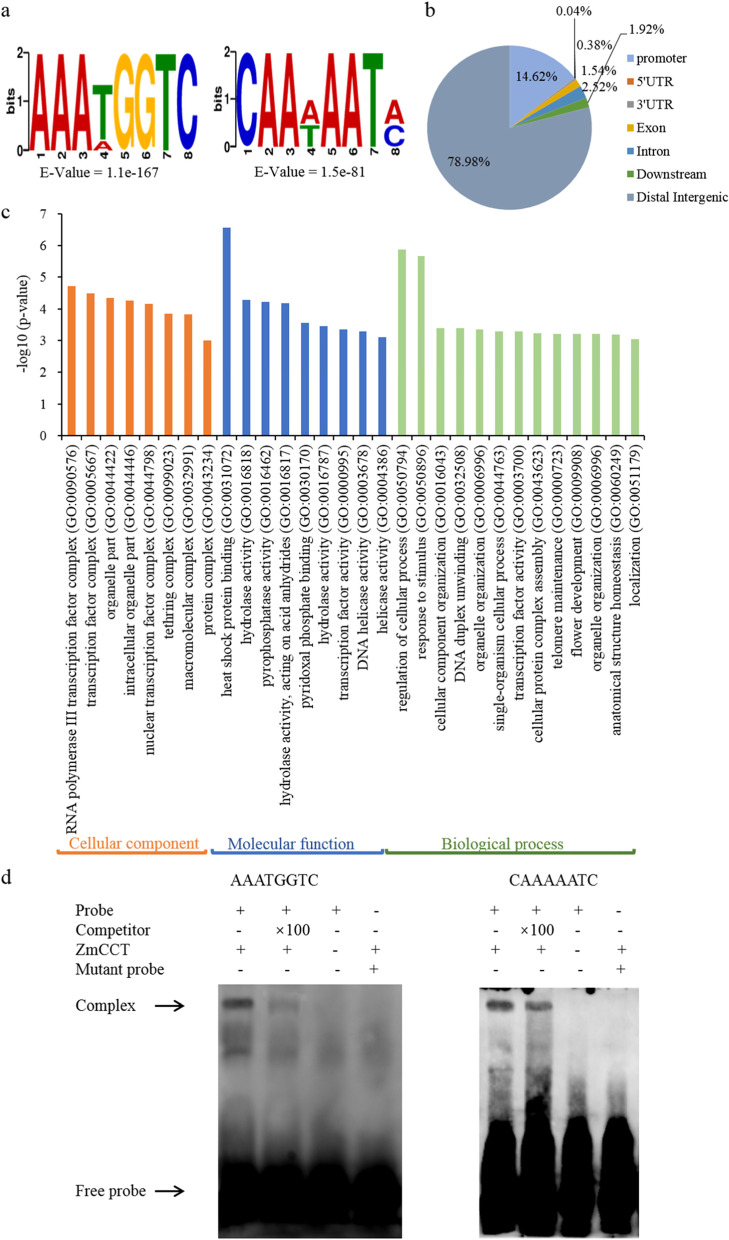
DAP-Seq analysis of *ZmCCT* target genes. **a)** *ZmCCT* binds to the AAATGGTC and CAAAAATC motifs as identified by the MEME-ChIP. **b)** Distribution of the *ZmCCT* binding sites in the maize V4 genome. **c)** GO annotation of targeted genes bound by ZmCCT protein. The y-axis represents the percentage of genes related to each functional category. **d)** Results of EMSAs confirming the *ZmCCT* binding to the AAATGGTC and CAAAAATC motifs

DAP-Seq assay was employed to identify the potential target genes directly regulated by *ZmCCT*. Using the Illumina platform (PE150 sequencing strategy), the DAP-Seq assay produced about 30 million reads of two biological replicates. Out of those reads, about 21 million reads were uniquely mapped to the maize_V4 reference genome. The effective mapping ratio was about 72%. The MACS2 algorithm version 2.2.7.1 was employed to identify *ZmCCT* binding sites. A total of 15,862 peaks across the entire maize V4 genome in the two biological replicates are significantly associated with two motifs, i.e., AAATGGTC and CAAAAATC (Fig. 4a) (*p*-value < 0.05). From all detected peaks, about 21% (3133 peaks) were located to genic regions including the promoter (− 3 kb to TSS), 5’UTR, 3’UTR, intron and exon (TTS to 3 kb) (Fig. 4b). The 3133 peaks correspond to 2041 genes. GO enrichment analysis revealed that these genes are significantly enriched in the response to stimulus and flower development subcategories (Fig. 4c). Out of 2041 genes, *ZmCCT* is likely to bind to the promoter region of 1602 genes, and these genes are mainly involved in the cellular process (GO:0044763, *p* value = 5.1 × 10^− 4^), organelle organization (GO:0006996, *p* value = 4.5 × 10^− 4^), flower development (GO:0009908, *p* value = 6.2 × 10^− 4^), transcription factor activity (GO:0000995, *p* value = 4.5 × 10^− 4^) and response to stimulus subcategories (GO:0050896, *p* value = 2.1 × 10^− 6^).

To further validate the expression level of those genes as putative targets directly regulated by *ZmCCT*, we analyzed the overlapping genes between DEGs generated from the WT and OE-*ZmCCT* under LD conditions and putative target genes directly regulated by *ZmCCT* in the upstream regions. Twenty-one DEGs that are likely to be implicated in flowering time regulation, stress response and flower development were identified (Additional file 5). Out of those 21 genes, 11 genes were upregulated in the OE-*ZmCCT* plants, whereas the remaining 10 genes were downregulated.

### Binding motif analysis revealed novel *ZmCCT* cis-elements

The two novel DNA motifs AAATGGTC and CAAAAATC were selected as candidate binding sites by MEME-ChIP software (Fig. 4a). To confirm whether *ZmCCT* could bind to the AAATGGTC and CAAAAATC motifs, the electrophoretic mobility shift assay (EMSA) was performed using a purified recombinant ZmCCT protein and labeled DNA probes containing the *ZmCCT* binding sites, i.e., AAATGGTC and CAAAAATC. As shown in Fig. 4d, ZmCCT protein could bind to the AAATGGTC and CAAAAATC motifs. The addition of 100× unlabeled competitors reduced the detected binding sites of *ZmCCT*, and it could not bind to the mutated probes (ACGCTAGA and TCCGCGCT). In the absence of the ZmCCT protein, except for the free probe, no binding band was observed. These results confirmed the specific binding of the ZmCCT protein to the AAATGGTC and CAAAAATC motifs.

### *ZmCCT* directly regulates genes related to photoperiod-dependent flowering time

**Fig. 5:**
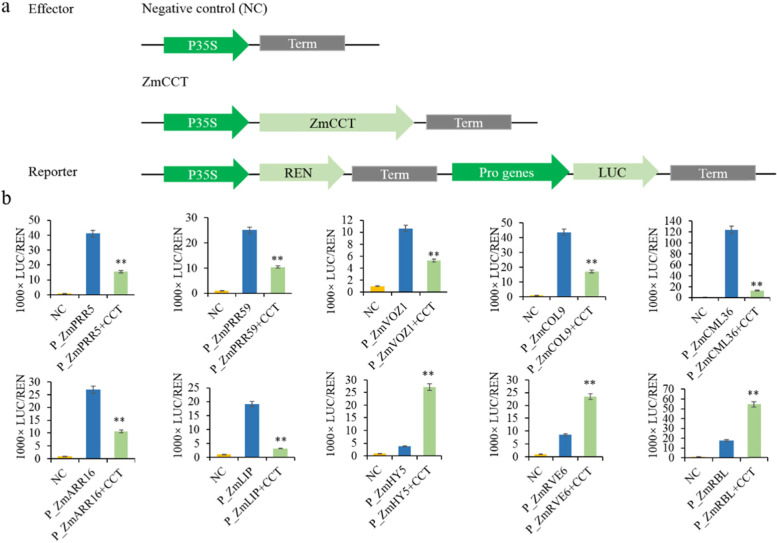
*ZmCCT* regulates a set of genes involved in maize photoperiod-dependent flowering time. **a)** The reporter construct was transiently expressed in *N. benthamiana* leaves together with the control vector or 35S::*ZmCCT* effector. Pro genes represent the promoters of the target genes. **b)** The LUC/REN ratio representing the relative activity of the gene promoters (n = 3, * *p* < 0.05; ** *p* < 0.01)

The DAP-Seq and RNA-Seq analyses identified 10 potential target genes of *ZmCCT* related to flowering regulation (Additional file 6), from which two *PSEUDO RESPONSE REGULATOR* (*ZmPRR5*), a *REVEILLE 6* (*ZmRVE6*) and a *CONSTANS-LIKE* (*ZmCOL9*). To investigate whether *ZmCCT* could regulate the expression of these potential target genes, a dual-luciferase transient transcriptional activity assay (referred to as Dual-LUC hereafter) in *N. benthamiana* leaves was carried out. The 35S::*ZmCCT* served as an effector and *LUC* (the firefly luciferase-coding gene) driven by different promoter regions (− 3000 to − 100 bp) of the potential target genes as a reporter (Fig. 5a). The results indicated that ZmCCT protein promotes the expression of three of the ten target genes, while represses the expression of the remaining seven genes (Fig. 5b). In particular, *ZmCCT* binds to the promoters of *ZmPRR5* (− 1567 bp), *ZmRVE6* (− 420 bp) and *ZmCOL9* (− 1315 bp) to promotes the expression of *ZmRVE6*, and represses the expression of *ZmPRR5* and *ZmCOL9* (Fig. 5b, Additional file 3, Fig. S2a).

### *ZmCCT* directly regulates a suite of stress-response genes

**Fig. 6:**
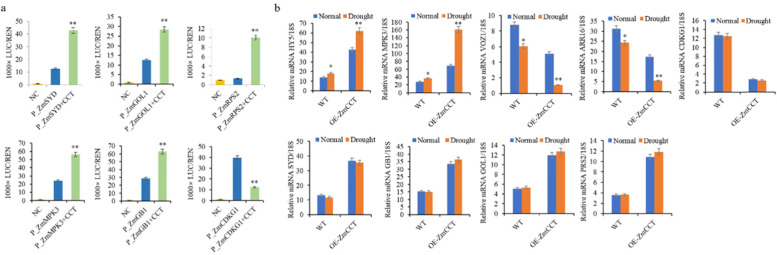
*ZmCCT* regulates a set of genes involved in stress response. **a)** The LUC/REN ratio representing the relative activity of the gene promoters. **b)** Expression analysis of the targeted genes in the WT and OE-*ZmCCT* plants at the V6 growth stage under drought and LD conditions by RT-qPCR (*n* = 3, * *p* < 0.05; ** *p* < 0.01)

RNA-Seq exhibited differential expression of nine genes associated with the plant response to biotic and abiotic stresses (Additional file 7),, from which six genes were upregulated and three were downregulated in the OE-*ZmCCT*. The Dual-LUC assay results confirmed that of the RNA-Seq (Fig. 5b, 6a, Additional file 7). Three of those nine genes, i.e., *ZmVOZ1*, *ZmARR16*, *ZmHY5*, and *ZmMPK3*, are known to be involved in drought stress response [30–33]. The results further showed that *ZmCCT* binds to the promoters of *ZmVOZ1* (− 927 bp) and *ZmARR16* (− 2438 bp) to repress their expression, and to the promoters of *ZmHY5* (− 2940 bp) and *ZmMPK3* (− 2841 bp) to promote their expression (Fig. 5b, 6a, Additional file 3, Fig. S2b). To further confirm that, we measured the mRNA levels of these nine genes by RT-qPCR in the WT and OE-*ZmCCT* plants between the well-irrigated and drought-stressed treatments under LD conditions. RT-qPCR results showed that the expression of *ZmHY5* and *ZmMPK3* was upregulated in the OE-*ZmCCT* and WT plants in response to drought stress, with the expression levels were much higher in the OE-*ZmCCT* compared to the WT plants (Fig. 6b). Meanwhile, *ZmVOZ1* and *ZmARR16* were downregulated in the OE-*ZmCCT* and WT plants after drought stress induction, and the expression levels were much lower in the OE-*ZmCCT* compared to the WT plants (Fig. 6b). The expression levels of the remaining 5 genes did not reveal any significant difference between the well-irrigated and the drought-stressed treatments (Fig. 6b). These results indicate that *ZmCCT* responds to drought stress primarily by direct regulation of the expression of those four drought-stress-related genes (*ZmHY5*, *ZmMPK3*, *ZmVOZ1* and *ZmARR16*).

### *ZmCCT* directly regulates several development-related genes

Six DEGs related to the developmental process were identified as direct targets of *ZmCCT* (Additional file 5). Four genes were upregulated and two genes were downregulated in the OE-*ZmCCT* plants. DAP-Seq results showed that *ZmCCT* has binding sites to the promoters of Zm00001d018977 (*ZmMET1*, − 1223 bp), Zm00001d037982 (*FAC*, − 2850 bp), Zm00001d034929 (*ZmLUG3*, − 2995 bp), Zm00001d044815 (*BOB1*, − 972 bp), Zm00001d008882 (*ZmM2*, − 2958 bp), and *ZmGB1* (− 1587 bp) (Additional file 5). These genes are involved in regulating plant growth and development.

## Discussion

Flowering time is an important agronomic trait that determines plant adaptation and distribution. The CCT domain-containing gene *ZmCCT* has been previously reported to play a negative role in regulating flowering time in maize [5, 6]. *ZmCCT* is a homolog to the rice photoperiod response regulator gene *Ghd7*. Enhanced expression levels of *Ghd7* under LD conditions delay heading date in rice [24]. Here we have employed several molecular techniques including DAP-Seq and RNA-Seq analyses to elaborate the molecular mechanisms of *ZmCCT* in photoperiod-dependent flowering time regulation and response to biotic/abiotic stresses in maize. Our data showed that *ZmCCT* negatively regulates maize flowering time and confers maize drought tolerance under LD conditions. The observed transcriptional activation and inhibitory activities displayed by *ZmCCT* when employing either the yeast and Gal4-LexA/UAS transcriptional systems might be due to the interaction with different transcription factors as the Yes-associated protein (YAP) and PDZ-binding motif (TAZ) do, which are known as oncogenic transcriptional co-activators and key regulators of stem cell function [34, 35]. Kim et al. [36] showed that YAP and TAZ could also function as transcriptional co-repressors when interacting with the TEA domain (TEAD) transcription factor. However, further studies are needed to explore the molecular mechanism of *ZmCCT*.

### *ZmCCT* delays flowering by regulating photoperiod-dependent flowering genes

The observed delayed flowering time in response to the overexpression of *ZmCCT* under LD conditions is consistent with previous studies [5, 6] where *ZmCCT*, a homolog of the rice photoperiod response regulator *Ghd7*, was consistently expressed at higher levels and confer delayed flowering in the teosintes under LD conditions. The DAP-Seq and RNA-Seq analyses results showed that *ZmCCT* directly promotes the expression of *ZmRVE6*, while represses the expression of *ZmPRR5* and *ZmCOL9*, resulted in a delayed flowering phenotype in maize. These findings suggest that *ZmCCT* delays flowering by upregulating the expression of a rhythmic gene *ZmRVE6* and downregulating the expression of the circadian oscillator gene *ZmPRR5* and a photoperiod-sensitivity gene *ZmCOL9*. These three genes (*ZmRVE6*, *ZmPRR5* and *ZmCOL9*) are involved in the photoperiod-dependent flowering time regulation and circadian rhythm in maize [37–40]. *ZmPRR5*, which is related to the domestication of maize, reduces the expression levels in the late-flowering phenotype *lfy1* mutant compared to the WT [37, 38]. *ZmRVE6* is a homolog of the Arabidopsis *REVEILLE 8 (AtRVE8*) that is involved in the photoperiodic flowering of Arabidopsis and shows a rhythmic expression in maize [39, 40]. Moreover, *RVE8* induced several evening-phased oscillator genes in Arabidopsis, including *PRR5* [41]. *ZmCOL9* belongs to C2C2-CO-like-transcription factor 5 and participates in the photoperiod-dependent teosinte flowering pathway [42, 43]. *ZmCOL9* is a homolog of the negative regulator of flowering in the photoperiod pathway in Arabidopsis *AtCOL9* [44], and *DTH2* that acts as an activator of rice heading under long-day conditions [45].

Moreover, RNA-Seq analysis revealed 10 DEGs between the WT and OE-*ZmCCT* plants under LD conditions. These genes belong to the circadian rhythm, photoperiodism regulation and flower development (Additional file 8), and comprise 3 CCT-domain-containing protein, 3 MADS transcription factor, *Adagio1*, *PHOT2*, *phyB2* and *GRAS53*. The expression levels of seven out of those ten genes are downregulated in the OE-*ZmCCT* plants. The circadian clock is an endogenous mechanism for keeping time, which allows organisms to coordinate biological processes and provide an adaptive advantage [46, 47]. our data showed that *ZmCCT* regulates the expression of *ZmHY5*, *ZmARR16*, *ZmCML36* and *ZmLIP*, which are related to the maintenance of the circadian period. More specifically, *ZmCCT* promotes the expression of *ZmHY5* but represses the expression of *ZmARR16*, *ZmCML36* and *ZmLIP* under LD conditions, which may prolong the circadian period thus delay flowering time. Under the blue light, a mutation in the *HY5* in Arabidopsis remarkably shortens the circadian period [48]. *AtHY5* affects the clock via the transcriptional repression of *PRR5* [48]. Homologs of the *ZmARR16* in Arabidopsis, i.e., *ARR3* and *ARR4*, are key genes for a proper circadian period and define an additional level of the circadian clock regulation [49]. *ARR3* or *ARR4* mutant could prolong the period of the clock regardless of the presence or absence of light [49]. In Arabidopsis, the cytosolic-free Ca^2+^ ([Ca^2+^]cyt) circadian oscillations could influence the function of the circadian clock via the Ca^2 + −^dependent action of *CALMODULIN-LIKE24* (*CML24*) [50]. The *cml23-2cml24–4* double mutant prolongs the circadian period [50]. The Arabidopsis homolog of the *ZmLIP* (*AtLIP1*) acts as a unique negative regulator in controlling the light input of the circadian clock, which is necessary for precise clock entrainment in plants. The *lip1–1* mutant shortens the circadian period by 1.5–2 h than the wild-type plants under continuous red light in Arabidopsis [51].

Taken together, our results suggest that *ZmCCT* could directly or indirectly regulate the expression of several photoperiod-dependent and circadian clock maintenance flowering time genes to affect maize flowering time.

### *ZmCCT* improves stress tolerance by regulating a set of stress response genes

Among the target genes of *ZmCCT*, several genes were shown to be associated with plant response to various biotic and abiotic stresses, which is consistent with a previous study where *ZmCCT* has been reported to be implicated not only in regulating flowering time but also in the stress response [25]. In particular, the overexpression of *ZmCCT* confers maize drought tolerance under LD conditions by regulating the expression of drought stress-responsive genes. After drought stress induction, *ZmHY5* and *ZmMPK3* expression in transgenic plants overexpressing the *ZmCCT* transgene was strongly elevated compared to the WT, suggesting that the effect of *ZmHY5* and *ZmMPK3* on drought stress is mediated through the promotion of *ZmCCT*. These results are consistent with previous studies where drought stress promotes the expression of *ZmHY5* and *ZmMPK3* [30, 31]. Similarly, the strong downregulation of the drought-responsive genes *ZmVOZ1* and *ZmARR16* in the OE-*ZmCCT* plants compared to the WT after drought induction suggests that *ZmCCT* mediates drought tolerance by suppressing the expression of *ZmVOZ1* and *ZmARR16*. A previous study showed that *ZmARR16* was downregulated in the ear, leaf and kernel of maize under drought stress [32]. The *voz1voz2* double mutant enhanced drought-stress tolerance in Arabidopsis [33]. Furthermore, *ZmCCT* may have a role in response to biotic stresses by regulating target genes. For instance, *ZmVOZ1* was downregulated in response to the infection with the rice black-streaked dwarf virus (RBSDV) [52]. Moreover, the expression of *ZmMPK3* was elevated in response to the infection with *Setosphaeria turcica* and necrotrophic fungi *Cercospora zeae-maydis* and *Cercospora Zeina* [53, 54]. Besides, the strong promoted expression of the defense and immune responsive genes *ZmSYD*, *ZmGB1* and *ZmRPS2* [55–59] in the transgenic plants carrying the *ZmCCT* transgene compared with WT suggests that *ZmCCT* plays a crucial role in maize response to biotic stresses.

### *ZmCCT* might be involved in regulating plant development

The identification of several *ZmCCT* target genes implicated in regulating plant growth and development, such as *ZmGB1*, *ZmLUG3* and *ZmM2* suggests that *ZmCCT* plays indirect roles in regulating plant development. *ZmGB1* encodes a heterotrimeric G protein β subunit and regulates shoot meristem development in maize [60]. ZmLUG3 protein belongs to the Gro/Tup1 family, which acts as negative transcriptional regulators and play important roles in the growth and developmental processes of many organisms [61]. *ZmM2* is involved in maize ear development and is regulated by the *BRANCHED SILKLESS 1* (*BD1*) [62]. The mRNA of *ZmM2* was not detected during the development of *bd1* ears [62]. Besides, *ZmCCT* regulates the expression of the Arabidopsis and rice homologs *ZmFAC*, and *ZmMET1* which are known to be involved in plant development [63–65]. ZmCCT may be involved in plant growth and development by regulating those development related genes.

**Fig. 7:**
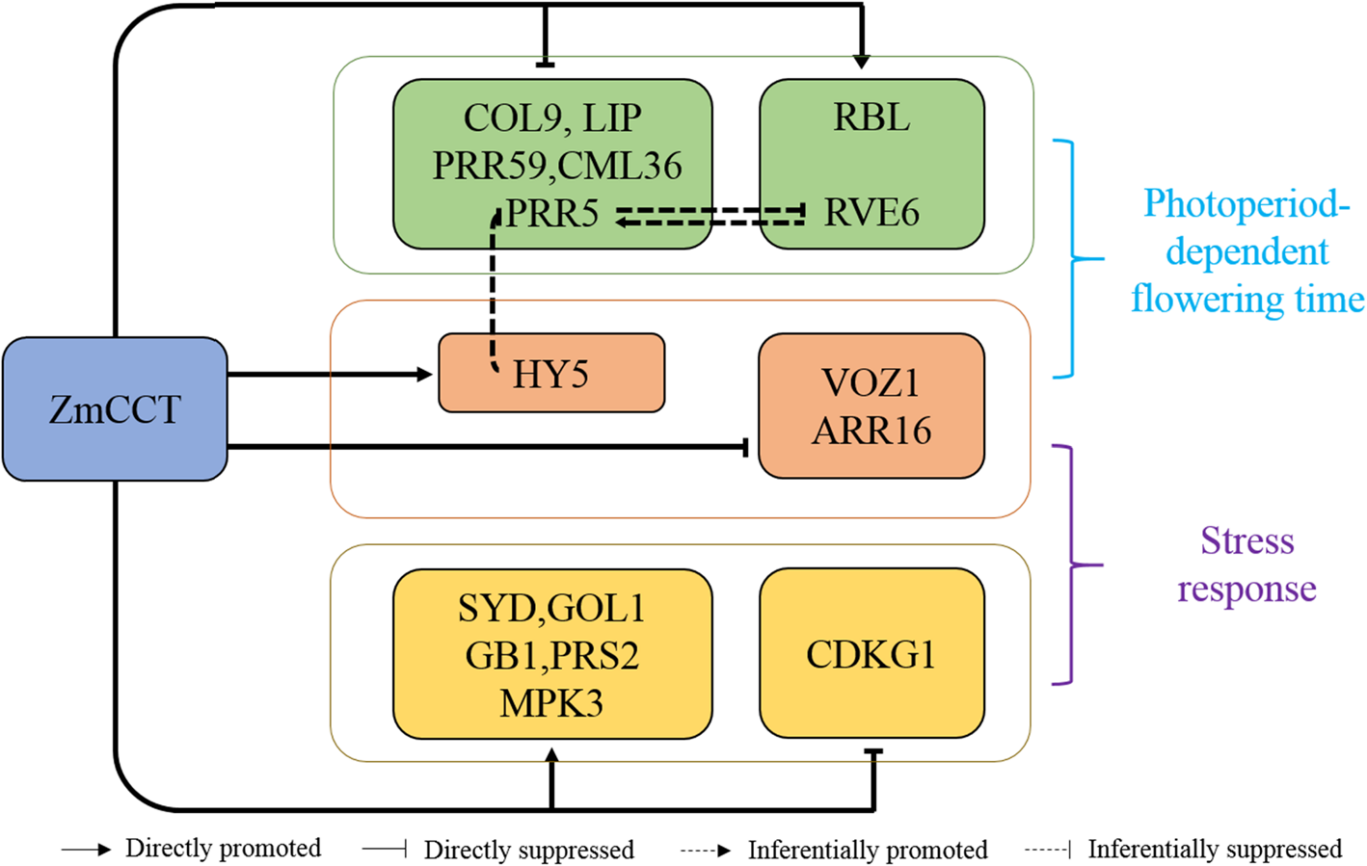
A regulatory model of the roles of *ZmCCT* gene in regulating flowering-time and stresses response in maize. T-bars indicate suppression. Dashed t-bars indicate inferentially suppressed. Arrows indicate positive regulation. Dashed arrows indicate inferentially promoted. Genes in the green boxes represent the target genes involved in the photoperiod-dependent flowering regulation. Genes in the yellow boxes represent the target genes involved in the stress response. Genes in the orange boxes represent the target genes that may have dual functions in regulating flowering time and response to stress

Accordingly, we proposed a regulatory model for the role of *ZmCCT* in regulating flowering time and stress response in maize (Fig. 7). Previous studies on the model plant Arabidopsis revealed that *ZmPRR5* and *ZmRVE6* may form a negative feedback loop in maize [40, 41]. Interestingly, three genes, i.e., *ZmHY5*, *ZmVOZ1* and *ZmARR16*, might have dual functions in regulating flowering time and stress response [30, 32, 33, 48, 49, 66]. Our data showed d that *ZmCCT* represses the expression of most flowering time genes, whereas promotes the expression of most stress-responsive genes. Two lines of evidence suggest that *ZmCCT* delays flowering by repressing flowering time-related genes and enhances stress tolerance by promoting the stress-responsive genes. Firstly, the RNA-Seq results showed a strong downregulation of seven of the ten DEGs related to flowering time in the transgenic plants overexpressing the *ZmCCT* transgene compared to the WT. Secondly, out of the identified 48 DEGs related to stress response, 40 genes were strongly upregulated in the transgenic plants overexpressing the *ZmCCT* compared to the WT.

## Conclusions

*ZmCCT* has dual functions in regulating maize flowering time and stress response. *ZmCCT* negatively regulates flowering time and enhances maize drought tolerance under LD conditions. *ZmCCT* delays flowering by repressing flowering time-related genes and enhances stress tolerance by promoting the stress-responsive genes.. Based on the results of this study, a model for the regulatory role of *ZmCCT* in flowering time and stress response in maize was proposed. Our results contribute to a comprehensive understanding of the regulatory mechanisms of *ZmCCT* in regulating flowering time and stress response in maize.

## Methods

### Plant materials and growth conditions

In this study, the full-length coding sequence of the *ZmCCT* was inserted into pCAMBIA1300-35S binary vector to overexpress *ZmCCT*. The wild-type (WT) B104 and *ZmCCT* overexpression transgenic (*OE-ZmCCT*) plants with B104 background were used. The B104 and Maize genetic transformation were performed by Beijing bomeixingao Technology Company. Seeds of the WT and OE-*ZmCCT* plants provided by Beijing bomeixingao Technology Company. The OE-*ZmCCT* and WT plants were grown in growth chambers (GR64, Conviron, Canada) under either long days (LD) and short days (SD) conditions. For LD conditions 15 h light/9 h dark, day temperature 28 °C, night temperature 22 °C, with a 40% relative humidity, a light intensity of 105 μmol m^− 2^ s^− 1^ in Zhengzhou, China, in the spring of 2018 were implemented. Meanwhile, under SD conditions, plants were subjected to 9 h light/15 h dark, other parameters were the same as for LD conditions. In this study, leaf blade tissue was collected from the leaves of the OE-*ZmCCT* or WT plants grown either under LD or SD conditions at the V6 growth stage. Each sample was collected from four different randomly selected plants. Three biological replicates were collected at the same time.

### RNA extraction and RNA sequencing (RNA-seq)

For each sample, total RNA was isolated using the RNeasy Plant Mini Kit (Qiagen), and then purified by magnetic stand (Invitrogen). The cDNA libraries were prepared using 5 μg of the total RNA following the Illumina standard protocol (TruSeq Standard RNA LT Guide). Twelve separate cDNA libraries were constructed. An Agilent 2100 Bioanalyzer system was employed to perform the quality control checks of all libraries. Qualified cDNA libraries were sequenced using the Illumina HiSeq 4000 system, and 150 bp paired-reads were generated.

### RNA-Seq data analysis

The RNA-Seq data analysis was performed according to the method described by Cao et al. [67]. To validate the differences in the expression levels observed by RNA-Seq between the WT and OE-*ZmCCT* plants under LD and SD conditions, we performed RT-qPCR on 20 differentially expressed genes. The primer sequences used in the RT-qPCR assay are listed in Additional file 2. All analyses were conducted with three technical and biological replicates.

### Transcriptional activation assay in yeast

To analyze the transcriptional activity of *ZmCCT*, the yeast strain AH109 (Stratagene, USA) that contains the *lacZ* and *HIS3* reporter genes was used. The coding sequence (CDS) of *ZmCCT* cloned from the maize inbred line CML288 was inserted into the pBD-GAL4 vector via *BamHI* and *NdeI* restriction enzymes to produce ZmCCT-GAL4 fusion protein. The negative control pBD-GAL4, positive control pGAL4 and pBD-ZmCCT plasmids were transfected into the AH109 cells. The transfected yeast cells were transferred to YPDA or SD/−Trp/−His medium and cultured at 30 °C for 3–5 days. The β-Galactosidase filter assay was performed to determine the β-galactosidase activity of the transfected yeast cells (PT3024–1).

### Gal4/UAS system assay

The reporter (UAS-GUS) and effector (VP16 and Gal4) constructs were previously described by Tiwari et al. [68]. The ZmCCT-GAL4 effector construct contains the full-length coding sequence of *ZmCCT* fused into the N-terminus of the Gal4 DNA-binding domain under the control of CaMV-35S promoter. To normalize the expression of the GUS reporter gene, the 35S-LUC construct was co-transformed as an internal control. GUS and firefly luciferase (LUC) enzymatic assays were carried out in the *N. benthamiana* leaves. Five days after subculture, protoplasts were isolated from the cells. Digestion of cell walls was performed in a solution containing 1% (w/v) cellulase Onozuka R-10 (Serva), 0.1% (w/v) pectinase (Sigma), 0.5% (w/v) Macerozyme RS (Serva), and 0.25 M mannitol for 2 h at room temperature. The polyethylene glycol (PEG) approach was employed to transform the isolated protoplasts with 20 μg DNA of the reporter and the effector constructs or the mock. The LUC substrate (Promega, Madison, WI) was prepared according to the manufacturer’s instructions. An aliquot of 10 μl of sample extract was mixed with 50 μl of the LUC substrate, and the Zylux FB15 luminometer (Fisher Scientific, Pittsburgh, PA) was then employed to measure the luciferase activity. The fluorometry was implemented to determine the GUS activities using 4-methylumbelliferyl glucuronide as a substrate. The activity of the reporter gene was represented as the ratio of GUS to luciferase activity. The data represent the average of three biological replicates.

### DNA affinity purification sequencing (DAP-Seq) experiments

DAP-Seq assay was carried out as described by O’Malley et al. [69]. The NEB Next® DNA Library Prep Master Mix set for Illumina kit (NEB #E6040S) was implemented to prepare the DAP-Seq gDNA library. The pFN19K Vector (cat#G184A, Promega) was employed to fuse the *ZmCCT* into the HaloTag. The TNT SP6 High-Yield Wheat Germ Protein Expression System (L3260, Promega) was then implemented to express the ZmCCT-HaloTag fusion protein. Magen HaloTag Beads (G7281, Promega) was used to purify the fusion protein. The ZmCCT-HaloTag fusion protein and 500 ng of library DNA were co-incubated in 40 μl PBS buffer with a slow shaking for 1.5 h in a cold room. The beads were five-times washed in 200 μl PBS + NP40 (0.005%). The supernatant was discarded and an aliquot of 25 μl of elution buffer was added. Finally, beads were incubated at 98 °C for 10 min to elute DNA fragments. According to the fragment size of the library, the DAP-Seq library concentration for a given read count was measured. The mock DAP-Seq libraries used as a negative control were prepared as previously described except for adding protein to the beads.

### DAP-Seq data analysis

The clean reads were aligned to the maize_V4 reference genome using the software Bowtie 2 version 2.3.4.3 at the default parameters [70]. The MACS2 algorithm (Model-Based Analysis of ChIP-Seq) version 2.2.7.1 was implemented to identify peaks with BAMPE mode [71]. Genes that contain peaks located within 3 kb upstream the TSS (transcription start site) or downstream TTS (transcription termination site) were defined as target genes of the *ZmCCT*.

### Gene ontology (GO) enrichment analysis

GO enrichment analysis was performed using the AgriGO analysis toolkit version 2.0 [28]. According to maize GAMER, the GO annotations of maize B73_V4 protein-coding genes were used as a reference [72]. The type of GO in agriGO v2.0 was set as Plant GO slim. Fisher’s exact test was employed to determine the significance levels. The BY procedure in the agriGO v2.0 toolkit was implemented for multiple test corrections [73]. Results exhibited an FDR ≤ 0.05 were considered as significantly enriched GO terms.

### Electrophoretic mobility shift assay (EMSA)

The probes used for EMSA were labeled and annealed under the guidance of DIG Gel Shift Kit standard procedure (Roche). All binding reaction components were thawed on ice. For a binding reaction of 20 μl, 4 μl of binding buffer, 1 μl of poly [d (I-C)] (1 μg/μl), 1 μl of poly L-lysine (0.1 μg/μl), 2 μl labeled probe (0.4 ng/μl), 1 μl purified *ZmCCT* fusion protein ((25–75 ng/μl) and double distilled water were added. After mixing the mixture carefully, it was incubated for 15 min at room temperature. A native polyacrylamide gel of 6–8% acrylamide in 0.5× TBE buffer was prepared, and the binding reaction was loaded onto the gel and run in 10 cm × 10 cm × 0.1 cm PAGE at 80 V. After electrophoresis, an equilibrated nylon membrane was carefully placed onto the gel for 30 min at 400 mA to transfer the binding reaction. The membrane was then placed on a development folder or hybridization bag and 1 ml CSPD working solution was applied. After incubation at 37 °C for 10 min to enhance the luminescent reaction, images were taken.

### Transient assays for in vivo activation activity

For the dual-luciferase (Dual-LUC) assay, a fragment of about 2500 bp of the promoter region of the potential target gene was cloned into pGreenII0800-LUC to develop Pro::*LUC* reporter. The full-length coding sequence of the *ZmCCT* was inserted into pCAMBIA1300-35S binary vector to generate the 35S::*ZmCCT* effector. The Dual-LUC assay was carried out in *N. benthamiana* leaves. After injection, plants were grown at 25 °C with a 14 h/10 h light/dark cycle. The protein was extracted 48 h after injection (Cat# E1910, Promega). The GloMax®20/20 Luminometer (Cat# E5311, Promega) was used to measure the LUC activity. An aliquot of 100 μl of Stop and Glow Buffer was then added to the reaction before measuring the Renilla luciferase (REN) activity. Each sample has three biological replicates.

### Drought stress experiment

For drought stress experiments, two OE-*ZmCCT* lines were used. The OE-*ZmCCT* and WT plants were grown for 2 weeks in pots filled with garden soil under LD conditions. A well-irrigated (WI) and drought-stressed (DS) trials were conducted. The WI trial was irrigated with about 500 ml water per pot each day. The drought-stressed (DS) trial was also irrigated as the WI trial for 14 days, and then irrigation has been withheld [74]. The relative water contents (RWC) were measured in the OE-*ZmCCT* and WT plants to identify phenotypic variations. The RWC was calculated as essentially described in Kwasniewski et al. [75]. Leaves from the OE-*ZmCCT* and WT plants were sampled for RNA extraction. Each sample was collected from three different randomly selected plants. Data shown as an average of three biological replicates.

### Real-time reverse transcription PCR (RT-qPCR)

Total RNA was extracted using PureLink™ RNA Mini Kit (12183018A, ThermoFisher). An aliquot of 1.5 μg RNA was reverse transcribed using the Hifair® III 1st Strand cDNA Synthesis SuperMix for RT-qPCR (YEASEN). following the manufacturer’s procedure. RT-qPCR was performed using the SYBR® Green PCR Master Mix (ThermoFisher) on a LightCycler® 480II Sequence Detection System. Relative gene expression was measured according to the 2–ΔΔCt approach. The 18S ribosomal gene was selected to normalize gene expression for RT-qPCR. The primer sequences used in the RT-qPCR assay are listed in Additional file 2.

## Supplementary Information

**Additional file 1.** (XLS 26 kb)

**Additional file 2.** (XLS 30 kb)

**Additional file 3.**

**Additional file 4.** (XLS 30 kb)

**Additional file 5.** (XLS 29 kb)

**Additional file 6.** (XLS 26 kb)

**Additional file 7.** (XLS 27 kb)

**Additional file 8.** (XLS 33 kb)

## Data Availability

The raw data can be accessed from the NCBI Sequence Read Archive (SRA) platform under the accession number PRJNA727729 (http://www.ncbi.nlm.nih.gov/bioproject/727729).

## Abbreviations

DAP-Seq: DNA affinity purification sequencing
EMSA: The electrophoretic mobility shift assay
GO: Gene Ontology
LUC: Firefly luciferase
Dual-Luc: Dual-luciferase transient transcriptional activity assay
RWC: Relative water content
