# Supplementary material for: ZmCCT regulates photoperiod-dependent flowering and response to stresses in maize
Source: BMC Plant Biol. 2021 Oct 6;21:453. doi: 10.1186/s12870-021-03231-y (PMC8493678; doi:10.1186/s12870-021-03231-y)
Supplement: Supplementary file 3 — Additional file 3. [file 12870_2021_3231_MOESM3_ESM.docx]

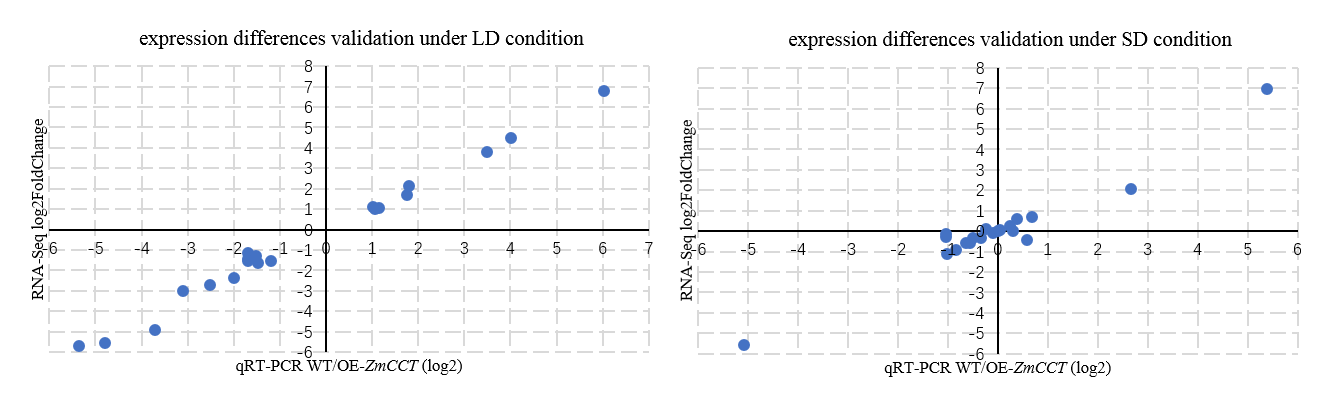


Fig. S1. Validation of the differential expression observed by RNA-Seq through RT-qPCR of 20 DEGs.


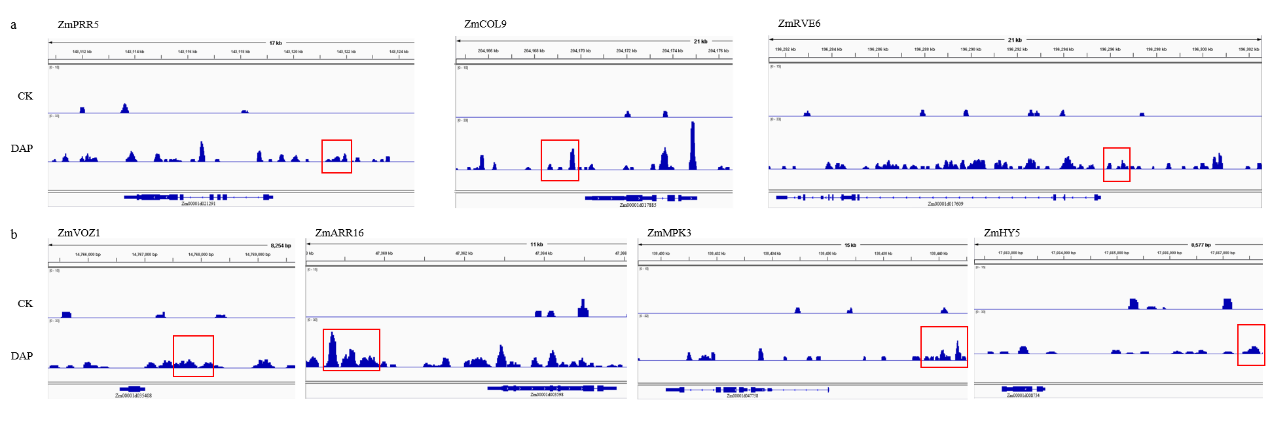


Fig. S2. Binding sites of the target genes of *ZmCCT* identified by DAP-Seq.

a) *ZmCCT* binding peaks of the flowering time target genes as shown in the Integrative Genomics Viewer. b) *ZmCCT* binding peaks of the stress response target genes as shown in the Integrative Genomics Viewer. The red box represents the location of *ZmCCT* binding peaks of the target gene
